# Metabolically stable bradykinin B2 receptor agonists enhance transvascular drug delivery into malignant brain tumors by increasing drug half-life

**DOI:** 10.1186/1479-5876-7-33

**Published:** 2009-05-13

**Authors:** Hemant Sarin, Ariel S Kanevsky, Steve H Fung, John A Butman, Robert W Cox, Daniel Glen, Richard Reynolds, Sungyoung Auh

**Affiliations:** 1 National Institute of Biomedical Imaging and Bioengineering, National Institutes of Health, Bethesda, Maryland 20892, USA; 2 Radiology and Imaging Sciences Program, Clinical Center, National Institutes of Health, Bethesda, Maryland 20892, USA; 3 Neuroradiology Department, Massachusetts General Hospital, Boston, Massachusetts 02114, USA; 4 Scientific and Statistical Computing Core, National Institute of Mental Health, Bethesda, Maryland 20892, USA; 5 Biostatistics, National Institute of Neurological Disorders and Stroke, National Institutes of Health, Bethesda, Maryland 20892, USA

## Abstract

**Background:**

The intravenous co-infusion of labradimil, a metabolically stable bradykinin B2 receptor agonist, has been shown to temporarily enhance the transvascular delivery of small chemotherapy drugs, such as carboplatin, across the blood-brain tumor barrier. It has been thought that the primary mechanism by which labradimil does so is by acting selectively on tumor microvasculature to increase the local transvascular flow rate across the blood-brain tumor barrier. This mechanism of action does not explain why, in the clinical setting, carboplatin dosing based on patient renal function over-estimates the carboplatin dose required for target carboplatin exposure. In this study we investigated the systemic actions of labradimil, as well as other bradykinin B2 receptor agonists with a range of metabolic stabilities, in context of the local actions of the respective B2 receptor agonists on the blood-brain tumor barrier of rodent malignant gliomas.

**Methods:**

Using dynamic contrast-enhanced MRI, the pharmacokinetics of gadolinium-diethyltriaminepentaacetic acid (Gd-DTPA), a small MRI contrast agent, were imaged in rodents bearing orthotopic RG-2 malignant gliomas. Baseline blood and brain tumor tissue pharmacokinetics were imaged with the 1^st ^bolus of Gd-DTPA over the first hour, and then re-imaged with a 2^nd ^bolus of Gd-DTPA over the second hour, during which normal saline or a bradykinin B2 receptor agonist was infused intravenously for 15 minutes. Changes in mean arterial blood pressure were recorded. Imaging data was analyzed using both qualitative and quantitative methods.

**Results:**

The decrease in systemic blood pressure correlated with the known metabolic stability of the bradykinin B2 receptor agonist infused. Metabolically stable bradykinin B2 agonists, methionine-lysine-bradykinin and labradimil, had differential effects on the transvascular flow rate of Gd-DTPA across the blood-brain tumor barrier. Both methionine-lysine-bradykinin and labradimil increased the blood half-life of Gd-DTPA sufficiently enough to increase significantly the tumor tissue Gd-DTPA area under the time-concentration curve.

**Conclusion:**

Metabolically stable bradykinin B2 receptor agonists, methionine-lysine-bradykinin and labradimil, enhance the transvascular delivery of small chemotherapy drugs across the BBTB of malignant gliomas by increasing the blood half-life of the co-infused drug. The selectivity of the increase in drug delivery into the malignant glioma tissue, but not into normal brain tissue or skeletal muscle tissue, is due to the inherent porous nature of the BBTB of malignant glioma microvasculature.

## Background

The normal blood-brain barrier (BBB) of brain microvasculature[1,2] prevents the transvascular passage of small hydrophilic chemotherapy drugs[3] or gadolinium (Gd)-based MRI contrast agents into normal brain tissue [4]. In contrast to the normal BBB, the blood-brain tumor barrier (BBTB) of malignant brain tumor microvasculature is porous due to fenestrations and gaps. This permits the selective entry of small conventional chemotherapy drugs or contrast agents into malignant glioma tumor tissue[5]. The clinically observed selective contrast enhancement of malignant brain tumor tissue on MRI following the intravenous bolus of gadolinium (Gd)-diethyltriaminepentaacetic acid (DTPA)[6] is due to the transvascular passage of the contrast agent across the BBTB and transient accumulation within the extravascular tumor space[7,8].

Even though the inherent leakiness of the BBTB does allow for the selective transvascular passage of small conventional chemotherapy drugs, such as carboplatin, these drugs do not achieve sufficiently high concentrations within tumor tissue after systemic infusion[9]. Bradykinin B2 receptor agonists are vasodilator peptides that act on the G-protein coupled bradykinin B2 receptors expressed on the endothelial and smooth muscle cells of the microvasculature supplying most tissues and organs[10,11]. Although bradykinin B2 receptors are ubiquitously expressed, these receptors are over-expressed in malignant tumors [12-15]. Since the bradykinin B2 receptor agonist-mediated activation of these over-expressed receptors results in the greater activation of nitric oxide[16] and prostaglandin[17] pathways in tumor tissue than in normal tissues, it is thought that the bradykinin B2 agonists selectively increase drug delivery across the blood-brain tumor barrier of tumor microvasculature, and in the case of peripheral solid tumors, the blood-tumor-barrier [16-19].

The intravenous co-infusion of a metabolically stable bradykinin B2 receptor agonist, labradimil (lobradimil, RMP-7, Cereport)[20], has been shown to be effective at enhancing the transvascular delivery of carboplatin[21] and other small therapeutics [22-24] across the BBTB. Based on quantitative autoradiography data, the findings of the published literature suggest that the primary mechanism by which labradimil increases transvascular drug delivery is by temporarily and selectively increasing the transvascular flow rate across the BBTB[23,25,26]. This mechanism of action, however, does not explain why in the clinical trial setting, the adaptive dosing of carboplatin has consistently over-estimated the carboplatin dose required to achieve the target carboplatin exposure[27,28]. We reasoned that this could be a consequence of labradimil increasing the blood half-life, and thereby, the tumor tissue half-life of any concurrently administered small therapeutic or imaging agent. As such, agent accumulation would not be expected to occur in the extravascular space of tissues with continuous microvasculature, such as normal brain[1,2] and skeletal muscle tissues[29,30]; therefore, an increase in transvascular agent delivery into brain tumor tissue would be selective, *per se*, for brain tumor tissue.

Based on our reasoning, we investigated the systemic actions of labradimil, as well as other bradykinin B2 receptor agonists with a range of known metabolic stabilities, in context of the local actions of the respective B2 receptor agonists on the BBTB of rodent malignant gliomas. We hypothesized that intravenously infused bradykinin B2 receptor agonists would increase the blood half-life of Gd-DTPA in proportion to the known metabolic stabilities of the respective agonists. We predicted that this increase in the blood half-life of Gd-DTPA would be evident in brain tumor tissue as well as skeletal muscle tissue; however, Gd-DTPA extravasation would occur across only the porous microvasculature of brain tumor tissue, and not across the continuous microvasculature of skeletal muscle tissue. Furthermore, in this study we sought to detect tumor location and volume dependent differences in the transvascular accumulation of Gd-DTPA within the same brain tumor tissue both at baseline and during the systemic infusion of bradykinin B2 receptor agonists. It is well known that there are tumor volume and location dependent differences in the transvascular flow rate across BBTB at baseline[31,32] within the same brain, however the significance of these differences has not yet been established in context of the systemic actions of bradykinin B2 receptor agonists of a wide range of metabolic stabilities[33].

For this study dynamic contrast-enhanced MRI was used[34], instead of quantitative autoradiography, which historically has been used to characterize transvascular flow rate across the BBTB[31,35]. Although quantitative for the concentration of radioactive agent within the tumor tissue at the experimental endpoint, the major limitations of autoradiography are: (1) the inability to determine the exact shape of the vascular input function due to the limited frequency at which blood can be manually sampled, especially during the initial time points; (2) the inability to measure continuously the change in the tumor tissue concentration of radioactive agent during the experimental time period, and (3) the inability to acquire data at baseline and during treatment in the same animal. In contrast to autoradiography, with dynamic contrast-enhanced MRI it is possible to image, in the same animal, the pharmacokinetics of a contrast agent at baseline and then during treatment[34,36].

With dynamic contrast-enhanced MRI we imaged the pharmacokinetics of Gd-DTPA in the blood and tumor tissue of rodents bearing orthotopic RG-2 malignant gliomas. We measured the change in blood and tissue Gd signal intensity with dynamic contrast-enhanced MRI, and determined the blood and tissue Gd concentration by calculating the molar relaxivity (*r*_1_) of Gd-DTPA *in vitro*[37] and then the change in the longitudinal relaxivity (*R*_1_) before and after contrast agent infusion for each imaged volume element (voxel) *in vivo*[38]. We tested four bradykinin B2 agonists of different known metabolic stabilities, with bradykinin (BK) being the least metabolically stable and labradimil, a synthetic peptide, being the most metabolically stable[11,20].

Based on this dynamic contrast-enhanced MRI-based approach, we were able to measure the blood and tissue pharmacokinetics of the 1^st ^bolus of Gd-DTPA over the first hour. We were then able to re-measure, in the same animal, the blood and tissue pharmacokinetics of a 2^nd ^bolus of Gd-DTPA over the second hour, the initial 15 minutes of which either normal saline (NS) or a bradykinin B2 receptor agonist was being infused intravenously. We visually compared the Gd concentration curve profiles of blood and RG-2 glioma tumor tissue from the 1^st ^and 2^nd ^Gd-DTPA boluses, calculated tumor tissue vascular parameters (*K*^trans^, *v*_e_, and *v*_p_) for each Gd-DTPA bolus, and conducted a percent change-based statistical analysis of tumor tissue vascular parameters as well as tumor and skeletal muscle tissue Gd-DTPA area under the concentration-time curve (AUC). We investigated bradykinin B2 receptor agonist treatment effects in the context of the volume of the RG-2 glioma and location of the RG-2 glioma being in either the anterior or posterior brain.

## Methods

### Bradykinin B2 agonists and preparation for infusion

Bradykinin B2 receptor agonist peptides were synthesized based on the known amino acid sequences (Peptides International, Inc., Louisville, KY)[11,20]. The peptides were received and stored in powder form, in 3 to 5 mg aliquots, at -20°C, until used. Each peptide was dissolved in sterile phosphate buffered saline (pH 7.4) to the appropriate concentration for infusion at the time of each experimental session. The infusion concentration of the BK, lysine-bradykinin (Lys-BK), and methionine-lysine-Bradykinin (Met-Lys-BK) solutions was 200 μg/mL, and the rate of infusion was 0.04 μmol/kg/min[35,39]. The concentration of the labradimil solution was 6 μg/mL, and the rate of infusion was 1 μmol/kg/min[40]. All bradykinin B2 receptor agonists were infused for 15 minutes, with the infusion of each agonist beginning 2 to 3 minutes prior to the 2^nd ^Gd-DTPA bolus.

### *In vitro *magnetic resonance imaging for calculation of Gd-DTPA molar relaxivity

All MRI experiments were conducted using a 3.0 tesla MR scanner (Philips Intera; Philips Medical Systems, Andover, MA) equipped with a 7 cm solenoid radiofrequency coil (Philips Research Laboratories, Hamburg, Germany). Gd-DTPA (Magnevist, 500 mM gadopentetate dimeglumine salt; Bayer, Toronto, Canada) was diluted using PBS into 200 μL microfuge tubes at concentrations (*C*) of 0.00 mM, 0.25 mM, 0.50 mM, 0.75 mM and 1.00 mM. The microfuge tubes were secured in level and upright positions within a plastic container filled with deionized ultra pure water. The container was placed in the small animal coil and centered within a 3 tesla MR scanner (Philips Intera; Philips Medical Systems, Andover, MA). Gd signal intensity measurements were then taken using a series of *T*_1 _weighted spin echo sequences with identical *T*_E _intervals (10 ms) and different *T*_R _intervals (100 ms, 300 ms, 600 ms and 1200 ms). Using the measured Gd signal intensity, in addition to the known values for *T*_R _and *T*_E_, the longitudinal relaxivity (*R*_1_,1/*T*_1_) and equilibrium magnetization (*M*_0_) were determined by non-linear regression (Eq. 1)[41].

(1)

The molar relaxivity (*r*_1_) was calculated by linear regression (Eq. 2)[41].

(2)

The molar relaxivity of Gd-DTPA was measured to be 4.05 1/mM*s. The relaxivity of Gd-DTPA calculated *in vitro *was assumed to be equivalent to the relaxivity of Gd-DTPA *in vivo *for the purposes of this study[37,42].

### Brain tumor induction and MRI suite set-up

All animal experiments were approved by the National Institutes of Health Clinical Center Animal Care and Use Committee. Cryofrozen pathogen-free RG-2 glioma cells were obtained from the American Type Culture Collection (Rockville, MD) and cultured in sterile DMEM supplemented with 10% FBS and 2% penicillin-streptomycin in an incubator set at 37°C and 5% CO_2_. The anesthesia and route for all animal experiments was isoflurane by inhalation with nose cone, 5% for induction and 1 to 2% for maintenance. On experimental day 0, the head of anesthetized adult male Fischer 344 rats (F344) weighing 200–250 grams (Harlan Laboratories, Indianapolis, IN) was secured in a stereotactic frame with ear bars (David Kopf Instruments, Tujunga, CA). The right anterior caudate and left posterior thalamus locations within the brain were stereotactically inoculated with RG-2 glioma cells[38,43]. In each location, either 20,000 or 100,000 glioma cells in 5 μL of sterile PBS were injected over 8 minutes, using a 10 μL Hamilton syringe (Hamilton Company, Reno, NV) with a 32-gauge needle[38].

On experimental days 11 to 12, the rats were re-anesthetized. Cannulation of both femoral veins and one femoral artery with polyethylene tubing (PE-50; Becton-Dickinson, Franklin Lakes, NJ) was performed and 40 cm long cannulas filled with heparinized normal saline (10 u heparin sodium/1 mL saline) inserted. To maintain a closed system, each cannula was connected to a 10 mL Luer-Lok plastic syringe (Becton-Dickinson Medical, Franklin Lakes, NJ), which also contained heparinized normal saline. One venous cannula was used for infusion of Gd-DTPA, and the other venous cannula was used for infusion of either NS or respective bradykinin B2 receptor agonist. The arterial cannula was used for blood pressure monitoring. 50 μL of blood was withdrawn from a venous cannula for measurement of hematocrit (Hct).

For imaging, the animal was transported to the 3 tesla Philips Intera MRI scanner, positioned in the solenoid small animal MRI coil, and a low pressure respiratory monitor (BIOPAC Systems, Inc., Goleta, CA) was placed around the animal's chest and loosely fastened with porous medical PE tape (Full Aid Company, Shanghai, China) to the edges of the gurney for the small animal MRI coil. During the initial set-up, two NS pre-filled 3 mL Luer-Lok plastic syringes (Becton-Dickinson Medical, Franklin Lakes, NJ) had been loaded onto separate micro-infusion pumps (PHD 2000; Harvard Apparatus, Holliston, MA) located in the MRI control room. In addition to the two 3 mL syringes filled with NS, a third 3 mL syringe filled with either NS or respective bradykinin B2 receptor agonist was loaded onto a third Harvard micro-infusion pump. The two 3 mL pre-filled NS syringes were connected to NS filled PE-50 tubings, and the third 3 mL syringe, filled with either NS or a bradykinin B2 receptor agonist, was connected to PE-50 tubing containing either NS or the respective bradykinin B2 receptor agonist, being careful not to introduce any air into the set-up. The PE-50 tubings were tunneled from the MRI control room to the MRI scanner room through an opening within the wall between the two rooms. In the scanner room, the distal ends of the two NS filled PE-50 tubings designated to be Gd-DTPA infusion tubings, were each connected to an additional piece of PE-50 tubing containing a 0.10 mmol Gd/kg dose of Gd-DTPA. Then, the distal free end of each of the Gd-DTPA containing tubings was connected to a prong of a micro-Y-connector pre-filled with NS. The remaining free end of the micro-Y-connector was connected to the rat's femoral venous cannula. In the MRI scanner, in a similar fashion, taking care not to introduce any free air, the rat's second femoral venous cannula was connected to the PE-50 tubing containing either NS or a bradykinin B2 receptor agonist. Lastly, the distal end of the rat's femoral artery cannula was connected to the NS filled PE-50 tubing of the arterial blood pressure monitoring system. The mean arterial blood pressure was measured using a small animal arterial blood pressure transducer connected to the MP-35 BIOPAC Student Lab system (BIOPAC Systems, Inc., Goleta, CA) located in the control room.

### *In vivo *magnetic resonance imaging

For imaging, the animal was positioned supine, with face, head, and neck snugly inserted into a nose cone centered within the 7 cm small animal solenoid radiofrequency coil. Anchored to the exterior of the nose cone were three 200 μL microfuge tubes containing 0.00 mM, 0.25 mM and 0.50 mM solutions of Gd-DTPA to serve as standards for measurement of MRI signal drift over time. In some case cases MRI signal drift was observed, therefore these data were excluded from further analysis. Coronal, sagittal, and axial localizer scans were used in order to identify the coronal plane most perpendicular to the rat brain dorsum. After orienting the rat brain in the image volume, a fast spin echo *T*_2 _weighted anatomical scan was performed. Image acquisition parameters for the *T*_2 _scan were: repetition time (*T*_R_) of 6000 ms, echo time (*T*_E_) of 70 ms, image matrix of 256 by 256, and slice thickness of 0.5 mm (over-contiguous). In order to quantify contrast agent concentration during post imaging processing, two separate three dimensional fast field echo *T*_1 _weighted (3D FFE T1W) scans were performed, one at a 3° low flip angle (low FA) of and the other at a 12° high flip angle (high FA). Image acquisition parameters for both scans were: *T*_R _of 8.1 ms, *T*_E _of 2.3 ms, image matrix of 256 by 256, and slice thickness of 1 mm (over-contiguous). The low FA scan was performed over 1.67 min, without any contrast agent on board. The high FA scan was a multi-dynamic scan consisting of 360 or 375 individual dynamic scans. The entire brain volume was imaged over 20 seconds for each dynamic scan resulting in the high FA scan duration being 120 or 125 minutes. Gd-DTPA was infused as a slow bolus, over 1 minute, so that the blood pharmacokinetics of Gd-DTPA could accurately be measured, especially during the early time points. At the beginning of the high FA scan, three to five pre-contrast brain volumes were acquired to guarantee the integrity of the *T*_1 _map without contrast agent (*T*_10_). Following acquisition of the pre-contrast brain volumes, 0.10 mmol/kg Gd-DTPA was dispatched (1^st ^Gd-DTPA bolus), and then once again, at the 1 hour time point in the scan (2^nd ^Gd-DTPA bolus). The NS or respective bradykinin B2 receptor agonist infusion was begun at the 57 minute mark and lasted for 15 minutes. The 2^nd ^Gd-DTPA bolus was dispatched approximately 2.5 minutes after the start of the normal saline or respective bradykinin B2 receptor agonist infusion, to ensure that the saline or agonist was in circulation for at least 2 minutes prior to the arrival of the Gd-DTPA bolus. Total volume infused per animal, including that associated with the two Gd-DTPA boluses, was less than 1.2 mL.

### Dynamic contrast-enhanced MRI scan data post-processing

Image data were analyzed using the Analysis of Functional NeuroImages (AFNI; ) software suite[44]. Motion correction and volume registration were performed by registering each dynamic high FA volume to the low FA volume, with image alignment based on least squares minimization using 3dvolreg. After volume registration, a *T*_1 _without contrast (*T*_10_) map was generated, by using the low FA signal data and the mean of the dynamic scan signal data before the visualization of the first Gd-DTPA contrast bolus (Eq. 3)[41].

(3)

The mean *T*_10 _signal value was determined voxel-by-voxel and then this data was used as input for the pharmacokinetic modeling done in AFNI using 3dNLfim. Computing concentration curves was an internal set of steps, but the actual fitting was done against the MRI signal data. The *T*_1 _with contrast concentration was calculated voxel-by-voxel for each high FA dynamic scan after visualization of the 1^st ^Gd-DTPA contrast bolus (Eq. 3). Using the mean *T*_10 _signal value and *T*_1 _signal values in addition to the Gd-DTPA molar relaxivity value, which was measured *in vitro *to be 4.05 1/mM*s, the Gd signal space data set was converted to a Gd concentration space data set (Eq. 2). Subsequent data analyses were conducted on two separate truncated Gd concentration space multi-dynamic scan data sets, one multi-dynamic scan data set for the first hour (1^st ^Gd-DTPA bolus) and the other multi-dynamic scan data set for the second hour (2^nd ^Gd-DTPA bolus).

For each tumor, a whole tumor region of interest was drawn manually, based on the time at which maximal contrast enhancement first occurred following the 2^nd ^Gd-DTPA bolus injection. For each left temporalis muscle and normal brain, a standard spherical 8.5 mm^3 ^region of interest was drawn. Vascular input functions were generated by visually inspecting and selecting a few voxels within the superior sagittal sinus that had both physiologically reasonable *T*_10 _values (~1100 ms), and peak Gd concentrations (~1.0 mM) that were closest to the estimated volume of distribution of Gd-DTPA in a 250 gram rat with a blood volume of approximately 14 mL[45]. The 2 to 3 voxels selected for the first and second part of the experiment were not necessarily the same voxels. Blood Gd concentration (*C*_b_) was converted to plasma Gd concentration (*C*_p_) by correcting for the hematocrit of each rat (Eq. 4)[46].

(4)

Since our brain volume acquisition rate was once every 20 seconds and the known transit time of blood movement between an artery to a vein within the brain is approximately 5 seconds[47], we selected the vascular input function voxels from the superior sagittal sinus, a large caliber brain vein with limited partial volume averaging related attenuation of signal intensity, as well as minimal distortion of signal related to blood flow effects.

### Dynamic contrast enhanced MRI-based pharmacokinetic modeling of brain tumor vascular parameters

The kinetic parameters were computed voxel-by-voxel over the entire brain volume using the 3dNLfim. Each Gd-DTPA bolus-based Gd concentration curve time series was analyzed using pharmacokinetic modeling voxel-by-voxel. The 2-compartment 3-parameter model generalized kinetic model [48] was used to model voxel-by-voxel brain tumor vascular parameters, both during the 1^st ^Gd-DTPA bolus and, once again, during the 2^nd ^Gd-DTPA bolus when either normal saline or the respective bradykinin B2 receptor agonist was infusing. For calculation of brain tumor tissue vascular parameters during the 1^st ^Gd-DTPA bolus, no residual contrast correction was performed when modeling, as reflected in Eq. 5 [48], since *C*_p_(0) = 0 and *C*_t_(0) = 0. However, for the calculation of tumor tissue vascular parameters during the 2^nd ^Gd-DTPA bolus, a residual contrast correction was applied when modeling, as reflected in Eq. 5, since *C*_p_(0) ≠ 0 and *C*_t_(0) ≠ 0, due to the presence of residual contrast from the 1^st ^Gd-DTPA bolus at the time of the 2^nd ^Gd-DTPA bolus.

(5)

*K*^trans ^– volume transfer constant from vascular space to extravascular extracellular space[46] – index of the transvascular flow rate across the blood-brain tumor barrier

*v*_e _– fractional extravascular extracellular volume[46] – index of tumor extravascular extracellular space

*v*_p _– fractional plasma volume[46] – index of tumor vascularity

*C*_t _(0) is defined as initial concentration of contrast agent in tumor tissue

*C*_t _(*t*) is defined as concentration of contrast agent in tumor tissue at time point (*t*)

*C*_p _(0) is defined as initial concentration of contrast agent in plasma

*C*_p _(*t*) is defined as concentration of contrast agent in plasma at time point (*t*)

Constraints on the parameters were set between 0 and 1, calling on 100,000 iterations. The units were unitless for both *v*_e _and *v*_p_, and in per minute for *K*^trans^. Least squares minimizations were performed by implementing the Nelder-Mead Simplex algorithm. Approximately 10% of voxels per tumor, usually located in the region of the tumor periphery, did not generate physiological parameters, due to a low signal to noise ratio and limitations of the curve fitting algorithm. These tumor voxels were censored based on visual inspection of curve fits and parameter distribution. Along the same lines, temporalis skeletal muscle tissue and normal brain tissue voxels did not generate physiologic parameters.

### Dynamic contrast enhanced MRI-based calculation of area under the concentration-time curve

For calculation of the tumor AUC, each time series per censored tumor voxel per injection per rat was averaged together to make an average censored time series per rat, which was weighted based on each tumor's volume. All rats, except one, grew two gliomas. One rat in the labradimil treatment group only grew an anterior glioma and no posterior glioma. Since the 2^nd ^Gd-DTPA bolus time series for each rat required that the residual contrast from the 1^st ^Gd-DTPA injection be taken into consideration, an exponential decay term was subtracted from each voxel's 2^nd ^Gd-DTPA bolus time series. The AUC data was then computed for each Gd-DTPA bolus by trapezoidal integration. The left temporalis skeletal muscle AUC was calculated in an analogous manner, but all voxels were used for calculation, since no modeling was performed, and therefore, no temporalis muscle voxels were censored.

### Statistical analysis for pharmacokinetic modeling and area under concentration-time curve

For all statistical analyses, the two RG-2 gliomas per rat were treated as correlated. The covariance structure in the multivariate analysis of covariance (MANCOVA) was assumed to be an unknown covariance structure while using the Kenward-Roger degrees of freedom method. For the statistical analyses of pooled 1^st ^Gd-DTPA bolus vascular parameter data, an initial MANCOVA was used to screen for a tumor volume by tumor location interaction, and there was no tumor volume by tumor location interaction. Subsequent MANCOVAs showed that there were significant tumor volume effects for all of the baseline vascular parameters. For the *v*_p _vascular parameter, in addition to a significant tumor volume effect, there was also a significant tumor location effect.

Statistical analyses of percent change-based tumor vascular parameter data, as well as of the tumor and temporalis muscle AUC data, were performed to examine treatment effects. For these data, an initial MANCOVA was used to screen for interactions of treatment group by tumor location and treatment group by tumor volume. If there were no significant treatment group interactions, subsequent MANCOVAs were used to examine the treatment effects with tumor location and volume being covariates. For percent change tumor vascular parameter data, there were no significant treatment group interactions for the *v*_e _and *v*_p _vascular parameters. There was a significant treatment group by tumor location interaction for the *K*^trans ^vascular parameter. Therefore, for *K*^trans^, treatment effects on anterior and posterior brain gliomas were examined individually, using an analysis of covariance (ANCOVA) with tumor volume being a covariate.

Censored tumor AUC data and uncensored left temporalis AUC data were analyzed. For tumor AUC data, there was a significant treatment group by tumor location interaction. Treatment group effects for anterior and posterior brain gliomas were examined individually, using the ANCOVA model with tumor volume being a covariate. Treatment group effects for the left temporalis muscle were examined using an analysis of variance (ANOVA) model, since the volume and location of the muscle region of interest was constant across animals. P-values reported are adjusted values using Dunnett-Hsu adjustments for multiple post hoc comparisons of treatment effect. All statistical tests were two-sided and implemented in SAS (SAS Institute Inc., Cary, North Carolina) with α = 0.05.

## Results

### Baseline RG-2 glioma vascular parameters

By modeling the blood and brain tumor tissue Gd concentration curves of the 1^st ^Gd-DTPA bolus, with the 2-compartment 3-parameter generalized kinetic model[48], we calculated the baseline RG-2 glioma tissue vascular parameter values prior to intravenous bradykinin B2 agonist infusion. Based on this data we were able to establish the relationship between RG-2 glioma tumor volume, and the baseline transvascular flow rate (*K*^trans^) across the BBTB, fractional extravascular extracellular tumor volume (*v*_e_), and fractional plasma volume (*v*_p_). These baseline vascular parameter values also served as internal control values for our percent change-based statistical analysis of change in baseline RG-2 glioma vascular parameters during the intravenous infusion of different bradykinin B2 receptor agonists.

**Figure 1:**
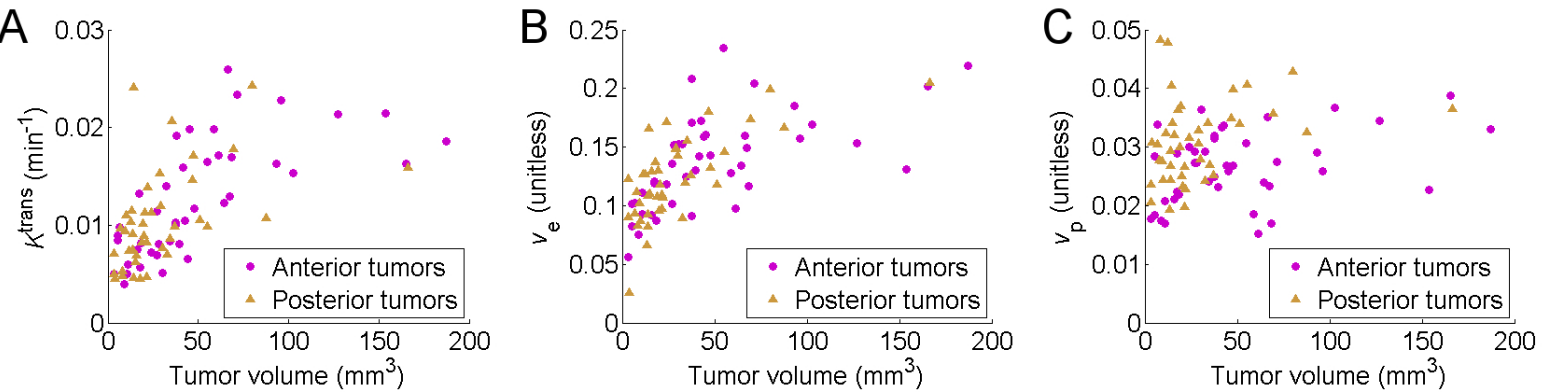
**Relationship between RG-2 glioma tumor location and volume and modeled baseline pharmacokinetic parameters**. (A) *K*^trans ^(transvascular flow rate, 1/min), (B) *v*_e _(extravascular extracellular space, fraction), (C) *v*_p _(vascular plasma volume, fraction). Anterior brain gliomas, N = 42; Posterior brain gliomas, N = 41.

We found that with an increase in RG-2 glioma tumor volume, there was also an increase in tumor tissue *K*^trans ^(F_1,66.4 _= 47.60, p < 0.0001), *v*_e _(F_1,75 _= 47.14, p < 0.0001), and *v*_p _(F_1,54.7 _= 10.79, p = 0.0018) (Figure 1A through 1C). RG-2 glioma location had no effect on tumor *K*^trans ^(F_1,44.3 _= 0.13, p = 0.7200) or *v*_e _(F 1,43.9 = 0.01, p < 0.9208). In the case of *v*_p_, an index of perfused tumor microvasculature, there was a tumor location effect, with RG-2 gliomas located within the posterior brain having a higher *v*_p _than those located within the anterior brain (F_1,43.3 _= 36.14, p < 0.0001) (Figure 1C).

### Mean arterial blood pressure during the infusion of bradykinin B2 receptor agonists

**Figure 2:**
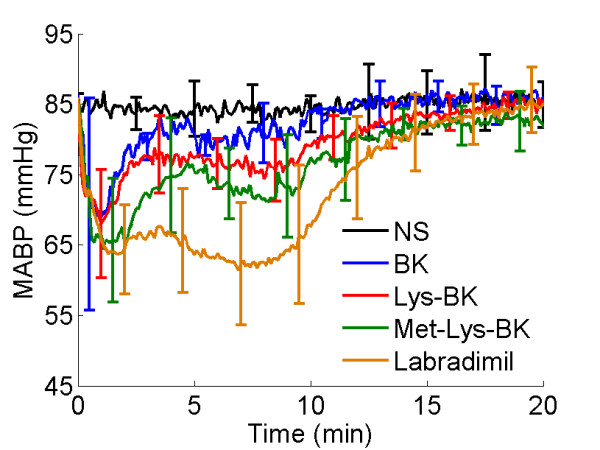
**Change in mean arterial blood pressure during the 15 minute intravenous infusion of normal saline or respective bradykinin B2 agonist**. NS, Normal Saline (N = 5); BK, Bradykinin (N = 5); Lys-BK, lysine-bradykinin (N = 7); Met-Lys-BK, methionine-lysine-bradykinin (N = 5); Labradimil (N = 11). Error bars represent standard deviation.

There was a decrease in mean arterial blood pressure during the intravenous infusion of each of the bradykinin B2 receptor agonists, as shown in Figure 2. The most significant fall in MABP was caused by the infusion of labradimil. However, both Met-Lys-BK and labradimil produced a similar initial decrease in MABP, which occurred during the first 2 to 3 minutes. In the case of Met-Lys-BK, the initial magnitude of fall in MABP did not persist. In the case of labradimil, it did persist and remained 10 to 15 mmHg lower than the decrease produced by Met-Lys-BK, before trending towards baseline (Figure 2).

### Blood half-life of Gd-DTPA as a result of the infusion of bradykinin B2 receptor agonists

**Figure 3:**
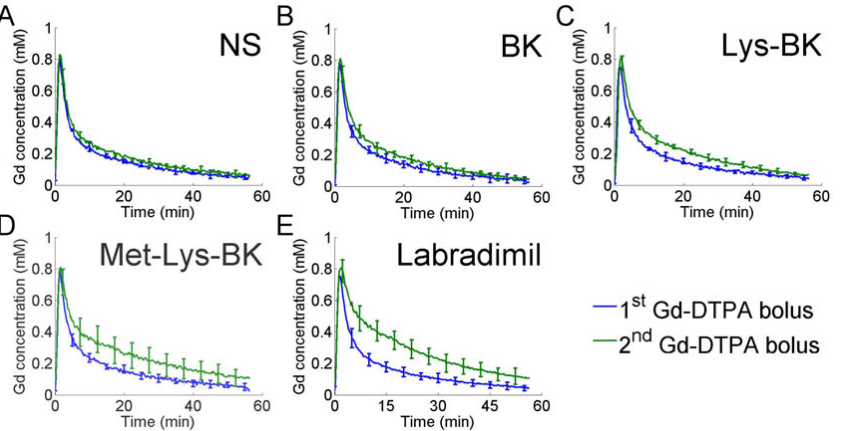
**Change in blood Gd concentrations of the 1^st ^Gd-DTPA bolus versus of the 2^nd ^Gd-DTPA bolus during 15 minute intravenous infusion of normal saline or respective bradykinin B2 agonist**. (A) NS (N = 6), (B) BK (N = 8), (C) Lys-BK (N = 8), (D) Met-Lys-BK (N = 7), (E) Labradimil (N = 13). Error bars represent standard deviation.

The change, over time, in blood Gd-DTPA concentration was measured in the superior sagittal sinus, which is a large caliber vein in the rat brain. The change in blood Gd-DTPA concentration for the 1^st ^hour of scanning, following the 1^st ^Gd-DTPA bolus, was compared to that over the 2^nd ^hour of scanning, following the 2^nd ^Gd-DTPA bolus. The 15 minute intravenous infusion of NS, beginning 2 to 3 minutes prior to the 2^nd ^Gd-DTPA bolus, had almost no effect on the blood half-life of Gd-DTPA, as evidenced by the similarities, over time, in the 1^st ^and 2^nd ^Gd-DTPA concentration curves in Figure 3, panel A. There was a slight increase in the blood half-life of Gd-DTPA with the intravenous infusion of BK (Figure 3B), and a somewhat greater increase with the infusion of Lys-BK (Figure 3C). The increase in blood half-life of Gd-DTPA was even greater with the infusion of Met-Lys-BK (Figure 3D). The greatest increase in blood half-life of Gd-DTPA was a result of the labradimil infusion (Figure 3E).

### Changes in transvascular flow rate across the BBTB due to the infusion of bradykinin B2 receptor agonists

Based on pharmacokinetic modeling of the 2^nd ^Gd-DTPA bolus concentration curve data and determination of the tumor vascular parameters during the intravenous infusion of either NS or bradykinin B2 receptor agonist, the percent change from baseline in the vascular parameters of anterior and posterior RG-2 glioma tumor tissues was calculated for each treatment group. By comparing the vascular parameter percent change of each bradykinin B2 receptor agonist group to that of the NS group, we found that there were no significant differences in *v*_e _(F_4,37.3 _= 1.91, p = 0.1300) and *v*_p _(F_4,36.5 _= 2.33, p = 0.0739). In the case of *K*^trans^, we found that there was a significant percent change in *K*^trans ^of the BBTB of anterior brain RG-2 gliomas (F_4,36 _= 11.62, p < 0.0001) and posterior brain RG-2 gliomas (F_4,35 _= 5.38, p = 0.0017) due to the infusion of bradykinin B2 receptor agonists. There was no statistically significant tumor volume effect on the change in *K*^trans ^in anterior brain gliomas (F_1,36 _= 3.49, p = 0.0698) as well as posterior brain gliomas (F_1,35 _= 2.31, p = 0.1378).

**Figure 4:**
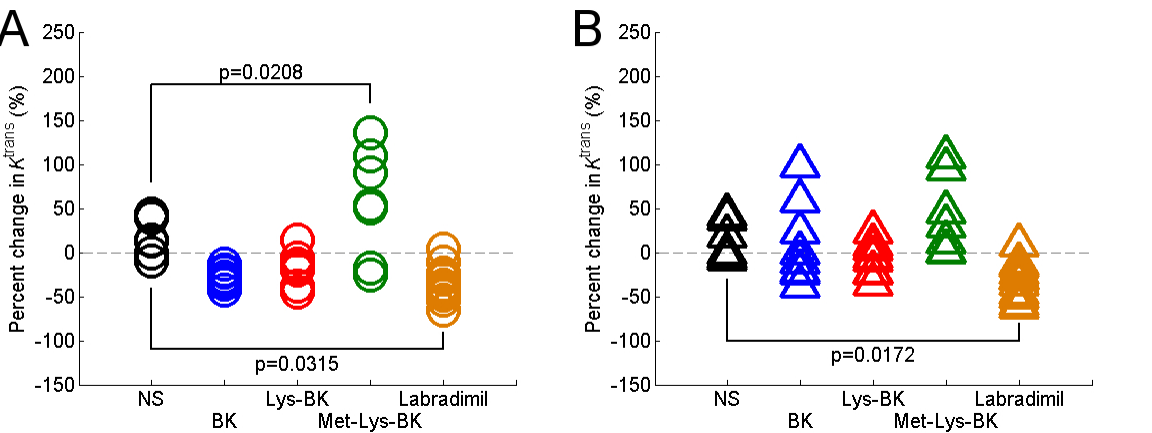
**Percent change in modeled *K*^trans ^of anterior and posterior brain RG-2 gliomas as a result of the 15 minute intravenous infusion of normal saline or respective bradykinin B2 agonist**. (A) Anterior brain RG-2 gliomas; NS (N = 6), BK (N = 8), Lys-BK (N = 8), Met-Lys-BK (N = 7), Labradimil (N = 13); (B) Posterior brain RG-2 gliomas; NS (N = 6), BK (N = 8), Lys-BK (N = 8), Met-Lys-BK (N = 7), Labradimil (N = 12). P-values reported are adjusted values using Dunnett-Hsu adjustments for multiple post hoc comparisons of treatment effect.

On post hoc analysis, in the BK group to NS group comparison, there was no significant change in *K*^trans ^of the BBTB for anterior brain (p = 0.1634) and posterior brain (p = 0.9978) RG-2 gliomas (Figure 4A and 4B). Likewise, in the Lys-BK group to NS group comparison, there was also no significant change in *K*^trans ^of the BBTB for anterior brain (p = 0.3260) and posterior brain (p = 0.6696) RG-2 gliomas (Figure 4A and 4B). In the Met-Lys-BK group to NS group comparison, there was a statistically significant percent increase in the *K*^trans ^of the BBTB in anterior brain RG-2 gliomas (p = 0.0208) (Figure 4A), but there was not a statistically significant increase in the *K*^trans ^of the BBTB in posterior brain RG-2 gliomas (p = 0.6049) (Figure 4B). In the labradimil group to NS group comparison, there was a statistically significant percent decrease in the *K*^trans ^of the BBTB for both anterior brain (p = 0.0315) and posterior brain (p = 0.0172) RG-2 gliomas (Figure 4A and 4B).

### Differences in pharmacokinetic behavior of Gd-DTPA in brain tumor and skeletal muscle tissues

**Figure 5:**
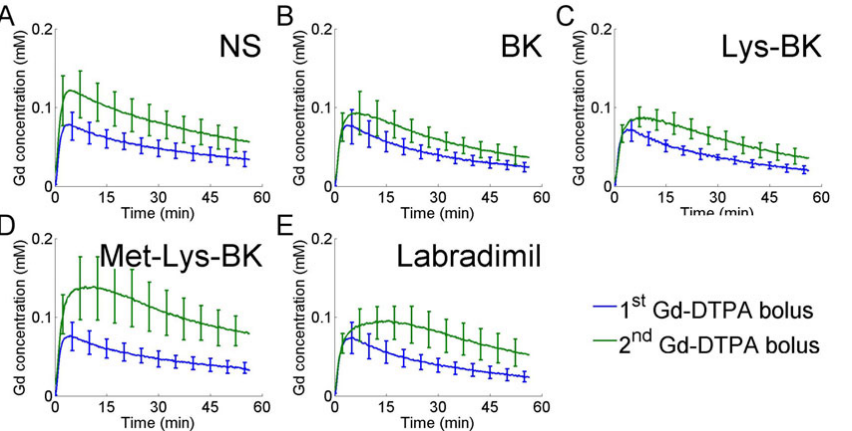
**Change in RG-2 glioma tumor tissue Gd concentrations of the 1^st ^Gd-DTPA bolus versus of the 2^nd ^Gd-DTPA bolus during 15 minute intravenous infusion of normal saline or respective bradykinin B2 agonist**. (A) NS (N = 6), (B) BK (N = 8), (C) Lys-BK (N = 8), (D) Met-Lys-BK (N = 7), (E) Labradimil (N = 13). Average tumor tissue concentration curves and standard deviation error bars are weighted with respect to total tumor volume within the respective treatment group.

The 1^st ^and 2^nd ^Gd-DTPA concentration curve profiles from RG-2 glioma tumor tissue, which has fenestrated microvasculature[49], were compared to those of temporalis skeletal muscle tissue, which has continuous microvasculature[30]. Both the 1^st ^and 2^nd ^Gd concentration curve profiles from RG-2 glioma tumor tissue (Figure 5A through 5E) did not mirror the respective Gd concentration curve profiles from blood (Figure 3A through 3E), since the Gd-DTPA extravasated out of the leaky tumor microvasculature and pooled in the extravascular tumor space. For ease of comparison, the blood and RG-2 glioma tumor tissue Gd concentration curves are shown together within a single figure in Additional file 1. As seen in the case of blood Gd-DTPA concentration curves, the degree of increase in half-life of Gd-DTPA in the extravascular tumor space correlated with the metabolic stability of bradykinin B2 agonist (Figure, 5A through 5E). As in blood, the increase in the half-life of Gd-DTPA in the extravascular tumor tissue space was greatest with labradimil infusion (Figure 5E).

**Figure 6:**
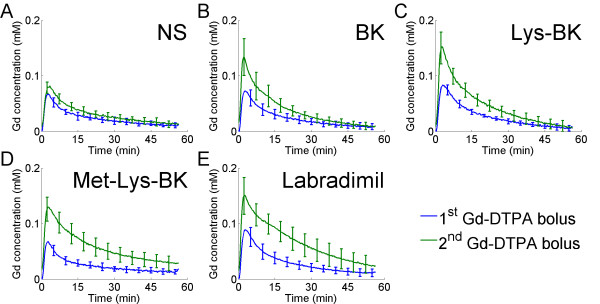
**Change in temporalis skeletal muscle tissue Gd concentrations of the 1^st ^Gd-DTPA bolus versus of the 2^nd ^Gd-DTPA bolus during 15 minute intravenous infusion of normal saline or respective bradykinin B2 agonist**. (A) NS (N = 6), (B) BK (N = 8), (C) Lys-BK (N = 8), (D) Met-Lys-BK (N = 7), (E) Labradimil (N = 13). Error bars represent standard deviation.

In contrast to the Gd-DTPA concentration curve profiles of RG-2 glioma tumor tissue, both the 1^st ^and 2^nd ^Gd concentration curve profiles from temporalis skeletal muscle tissue (Figure 6A through 6E) mirrored the respective Gd concentration profiles from blood (Figure 3A through 3E), since the Gd-DTPA remained predominantly within the skeletal muscle microvasculature, and did not extravasate into the extravascular tissue space. For ease of comparison, the blood and temporalis skeletal muscle tissue Gd concentration curves are shown together within a single figure in Additional file 2. There was an increase in the peak of the 2^nd ^Gd-DTPA concentration profile compared to the 1^st ^(Figure, 6B through 6E). This was not the case with NS infusion (Figure 6A), indicating that blood flow to skeletal muscle microvasculature increased with bradykinin B2 agonist infusion, irrespective of the metabolic stability of the agonist. As seen in the case of blood Gd-DTPA concentration curves of the superior sagittal sinus, the degree of increase in the half-live of Gd-DTPA within skeletal muscle tissue microvasculature correlated with the metabolic stability of the bradykinin B2 agonist (Figure 6A through 6E). As in blood of the superior sagittal sinus, the increase in the half-life of Gd-DTPA in skeletal tissue microvasculature was greatest with labradimil infusion (Figure 6E).

### Gd-DTPA area under the concentration-time curve in the brain tumor and skeletal muscle tissues

To quantify effect of increased Gd-DTPA half-life, for brain tumor and skeletal muscle tissues the percent change in Gd-DTPA AUC between the 1^st ^and the 2^nd ^Gd-DTPA concentration curve profiles. Comparisons of the percent change in Gd-DTPA AUC of each bradykinin B2 agonist group to that of the NS group were made.

**Figure 7:**
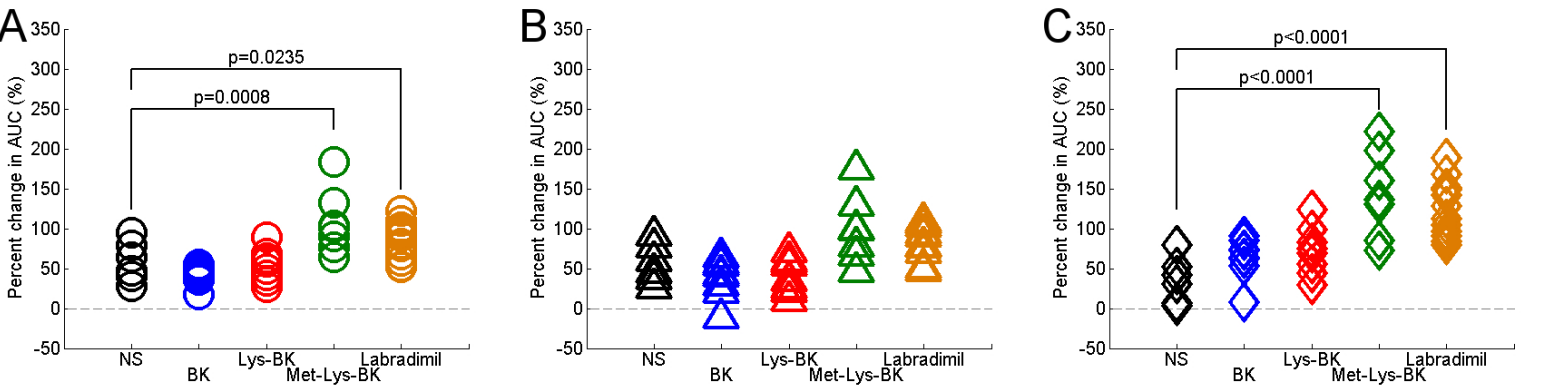
**Percent change in Gd-DTPA area under the time-concentration curve (AUC) of RG-2 glioma tumor tissue and temporalis skeletal muscle tissue as a result of the 15 minute intravenous infusion of normal saline or respective bradykinin B2 agonist**. (A) Anterior brain RG-2 gliomas; NS (N = 6), BK (N = 8), Lys-BK (N = 8), Met-Lys-BK (N = 7), Labradimil (N = 13); (B) Posterior brain RG-2 gliomas; NS (N = 6), BK (N = 8), Lys-BK (N = 8), Met-Lys-BK (N = 7), Labradimil (N = 12); (C) Temporalis skeletal muscle, NS (N = 6), BK (N = 8), Lys-BK (N = 8), Met-Lys-BK (N = 7), Labradimil (N = 13). P-values reported are adjusted values using Dunnett-Hsu adjustments for multiple post hoc comparisons of treatment effect.

In the case of brain tumor tissue, for anterior brain RG-2 gliomas there was significant percent change in Gd-DTPA AUC with bradykinin B2 receptor agonist infusion (F_4,36 _= 9.62, p < 0.0001), and there was a statistically significant tumor volume effect (F_1,36 _= 4.68, p = 0.0372), i.e. the percent change in Gd-DTPA AUC was dependent on the glioma tumor volume. For posterior RG-2 gliomas there was a significant percent change in Gd-DTPA AUC with bradykinin B2 receptor agonist infusion (F_4,35 _= 6.72, p = 0.0004), but no statistically significant tumor volume effect (F_1,35 _= 3.01, p = 0.0915). On post hoc analysis, in the BK group and Lys-BK group to NS group comparisons, there was no significant change in Gd-DTPA AUC for anterior brain and posterior brain RG-2 gliomas (Figure 7A and 7B). In the Met-Lys-BK group to NS group comparison, there was a significant percent increase in Gd-DTPA AUC for anterior brain (p = 0.0008) but not posterior brain (p = 0.0600) RG-2 gliomas (Figure 7A and 7B). Likewise, in the labradimil group to NS group comparison, there was a significant percent increase in Gd-DTPA AUC for anterior brain (p = 0.0235) but not posterior brain (p = 0.1286) RG-2 gliomas (Figure 7A and 7B). Since the post hoc analysis, in each of the bradykinin B2 receptor agonist group to NS group comparisons, did not reveal any significant differences in Gd-DTPA AUC, this indicates that there exists a significant difference in one or more other pair-wise comparisons, for example, in the in the Met-Lys-BK group and labradimil group to NS group comparisons.

In the case of temporalis skeletal muscle tissue, there was a significant percent change in Gd-DTPA AUC with bradykinin B2 receptor agonist infusion (F_4,37 _= 11.95, p < 0.0001). On post hoc analysis, in the BK group and Lys-BK group to NS group comparisons, there was no significant change in Gd-DTPA AUC (Figure 7C). In the Met-Lys-BK group to NS group comparison, there was a significant percent increase in Gd-DTPA AUC (p < 0.0001) (Figure 7C). Likewise, in the labradimil group to NS group comparison, there was a significant percent increase in Gd-DTPA AUC (p < 0.0001) (Figure 7C).

## Discussion

**Figure 8:**
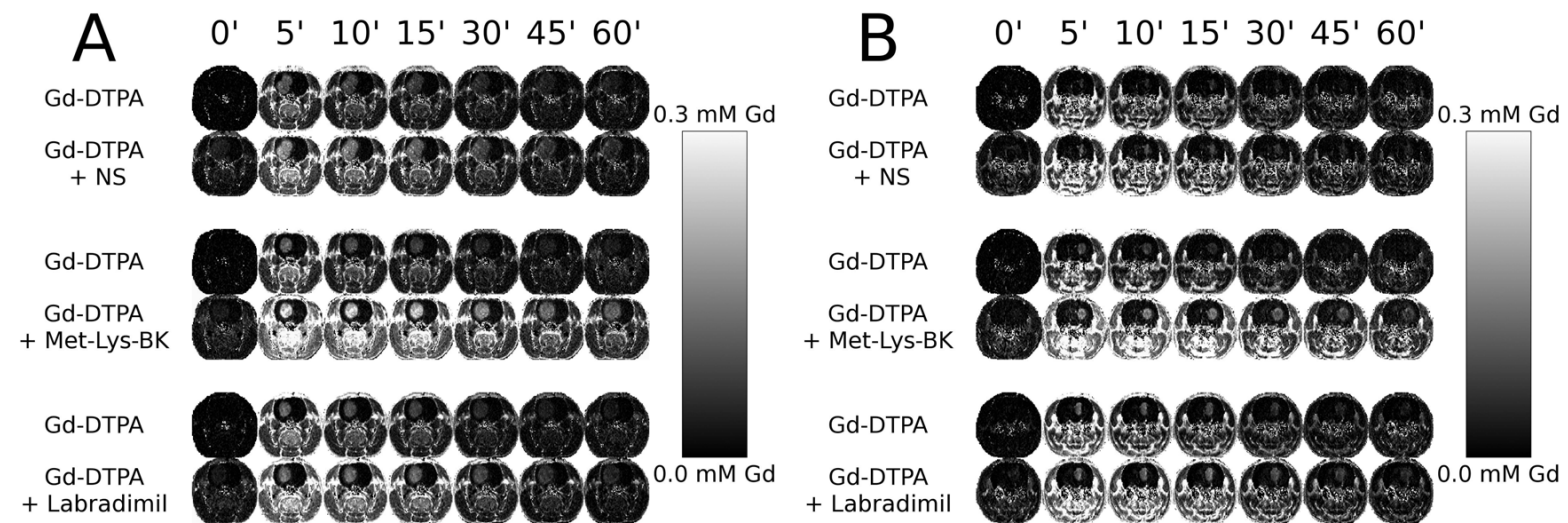
**Gd concentration maps over time of larger anterior brain RG-2 gliomas and smaller posterior brain RG-2 gliomas within a representative rat of the Normal Saline, Met-Lys-BK, and Labradimil groups**. (A) Anterior brain gliomas: tumor volumes, 153 mm^3 ^(NS), 127 mm^3 ^(Met-Lys-BK), 102 mm^3 ^(Labradimil); (B) Posterior brain gliomas: tumor volumes, 14 mm^3 ^(NS), 51 mm^3 ^(Met-Lys-BK), 35 mm^3 ^(Labradimil). Note: Residual contrast in tissue prior to the 2^nd ^Gd-DTPA bolus.

Historically, quantitative autoradiography has been used to determine how effective co-infused labradimil is at enhancing the transvascular delivery of a radioactive agent across the BBTB into tumor tissue[35]. Due to practical limitations in the frequency at which blood can be withdrawn from the subject during autoradiography, it is very difficult to determine accurately the continuous change in blood concentration of the radioactive agent and determination of the arterial input function[34]. Therefore, the autoradiography determination relies heavily the measurement of the amount radioactive agent in the harvested tumor tissue specimen, on the basis of which the unidirectional transfer constant, *K*_i_, is calculated[35]. Due to the unavailability of tumor tissue concentration curve data, an increase in the concentration of the radioactive agent in brain tumor tissue at the experimental endpoint would signify that the transvascular flow rate across the BBTB had increased during the infusion of labradimil, which has been the interpretation to date[23,25,26]. In this study, by using dynamic contrast-enhanced MRI, we were able to image during the 1^st ^hour, the blood and tissue pharmacokinetics of a bolus infusion of Gd-DTPA, and then, in the same animal head, re-image during the 2^nd ^hour the blood and tissue pharmacokinetics of a second bolus infusion of Gd-DTPA, during which either normal saline or a bradykinin B2 receptor agonist was infused for 15 minutes (Figure 8A and 8B). Data analysis of 2^nd ^Gd-DTPA bolus pharmacokinetics was conducted taking into account the decay of residual contrast related to the 1^st ^Gd-DTPA bolus, as detailed in the Methods section.

Although several dynamic contrast-enhanced MRI-based pharmacokinetic models exist, in this work we employed the 2-compartment 3-parameter generalized kinetic model since this model allows for the calculation of the fractional vascular plasma volume (*v*_p_), in addition to transvascular flow rate (*K*^trans^) and fractional extravascular extracellular space (*v*_e_)[46,48]. Using the generalized kinetic model, we modeled the 1^st ^Gd-DTPA bolus concentration curve data to determine the baseline RG-2 glioma tumor tissue vascular parameters. We found that the transvascular flow rate across the BBTB, extravascular extracellular space, and vascular plasma volume of RG-2 gliomas increased as RG-2 glioma tumor volume increased, regardless of whether the glioma was located in the anterior or posterior brain. These findings demonstrate that, as the volume of a brain tumor increases, the BBTB becomes more porous, the extravascular extracellular space enlarges, and the tumor becomes more vascular, and are in agreement with what has previously been reported for rodent brain tumors[32,33,50]. The posterior brain RG-2 gliomas in our study were located in the posterior thalamus of the rat brain. We found that these posterior thalamus gliomas had higher vascular plasma volumes than anterior caudate gliomas. This may be attributable to posterior brain tumors being in close proximity to choroid plexus of the rat brain ventricular cavities, as has been previously observed[50].

We interrogated the pharmacokinetics of Gd-DTPA during the intravenous infusion of normal saline, or a bradykinin B2 receptor agonist. The four bradykinin B2 receptor agonists, ranging from least to most metabolically stable were BK, Lys-BK, Met-Lys-BK, and labradimil. The additions of lysine and methionine to the amino terminus of Lys-BK (a decapeptide) and Met-Lys-BK (a hendecapeptide) respectively, confers resistance to degradation by blood aminopeptidases, compared to BK, which is an unmodified nonapeptide[11,51,52]. Labradimil is a nonapeptide like BK, but with unnatural amino acid substitutions at positions 3, 5, and 8, with a reduced peptide bond between positions 7 and 8. These modifications decrease the labradimil's rate of degradation by blood carboxypeptidases and angiotensin converting enzyme, and as a consequence, increase the peptide's blood half-life significantly compared to bradykinin, but this has been difficult to quantify[20,53]. However, these modifications also decrease its biological activity at the bradykinin B2 receptor compared to bradykinin[20,53].

Based on our measurements of the change in systemic mean arterial blood pressure (MABP) during the infusion of the respective bradykinin B2 receptor agonists, we show here that there is a clear association between the magnitude of decrease in MABP and metabolic stability of the respective bradykinin B2 receptor agonist, with labradimil's effect on reduction in MABP being more profound and persistent than that of the other bradykinin B2 receptor agonists. Since tumor microvasculature is known to lack autoregulatory capacity to maintain adequate blood flow when there is a significant decrease in MABP[54,55], with the fall in MABP we observed during the infusion of labradimil, there would likely be a reduction in blood flow to glioma tumor tissue. This has been shown to occur in rodent peripheral solid tumors during the intravenous infusion of labradimil[17].

After modeling the 2^nd ^Gd-DTPA bolus concentration curve data and calculating the percent change in baseline tumor tissue vascular parameters due to bradykinin B2 receptor agonist or NS infusion, we compared the percent change of each bradykinin B2 receptor agonist group to that of the NS group. The only vascular parameter to show a statistically significant difference due to bradykinin B2 receptor agonist infusions was *K*^trans^. We found that there was no statistically significant tumor volume effect on the percent change in *K*^trans ^for either anterior brain or posterior brain RG-2 gliomas. These findings suggest that observed changes in *K*^trans ^due the systemic infusion of bradykinin B2 agonists may be independent of RG-2 glioma tumor volume and location, and instead a reflection of bradykinin B2 receptor agonist-mediated systemic hemodynamic changes on local transvascular flow rate across the BBTB, irrespective of brain tumor volume and location.

The statistically significant increase in *K*^trans ^of the BBTB of anterior RG-2 gliomas that we observed with intravenous Met-Lys-BK infusion would be attributable to the combination of: (1) a higher affinity than labradimil for the bradykinin B2 receptors over-expressed on tumor microvasculature and thereby, greater ability to vasodilate tumor microvasculature and increase the permeability of the BBTB; and (2) the lesser metabolic stability than labradimil resulting in a less significant fall in MABP than that caused by labradimil infusion. Even though this is the first study to investigate changes in the transvascular flow rate across the BBTB with Met-Lys-BK, it has been shown in rabbit and guinea pig intradermal injection preparations that Met-Lys-BK is at least as potent as bradykinin in enhancing vascular permeability, and in some cases was shown to be more potent[52]. Furthermore, Met-Lys-BK is more resistant to inactivation by human, dog, and guinea pig plasma kininases compared to bradykinin[52]. In the context of the less significant fall in MABP produced by the infusion of Met-Lys-BK, as compared to labradimil, the *K*^trans ^of the BBTB would be expected to increase with the intravenous infusion of Met-Lys-BK. In general, with regards to the posterior brain RG-2 gliomas of the study tumor population, our inability to show statistical significance, if it existed, could be attributable to our limited image spatial resolution[56,57] for tumor volumes less than 25 mm^3^, which was the size range of more posterior brain tumors compared to anterior brain tumors (Additional file 3).

The statistically significant decrease in the *K*^trans ^of the BBTB of both anterior and posterior gliomas with labradimil infusion that we observed would be attributable to the combination of: (1) the greater metabolic stability than Met-Lys-BK that resulted in a more significant fall in MABP as compared to that caused by Met-Lys-BK infusion, and (2) a lower affinity than Met-Lys-BK for the bradykinin B2 receptors over-expressed on tumor microvasculature. The dramatic and prolonged fall in MABP caused by labradimil infusion is expected to reduce blood flow to glioma tumor tissue, since tumor microvasculature lacks the autoregulatory capacity to maintain adequate blood flow when there is a significant decrease in MABP[54,58], as has been shown in rodent peripheral solid tumors[17]. In addition, in a blood flow-limited state, a decrease in *K*^trans ^modeled based the pharmacokinetics of a small MRI contrast agent, such as Gd-DTPA, would signify a decrease in tumor blood flow than a decrease in transvascular flow rate across the BBTB[59]. Therefore, the decrease in *K*^trans ^of the BBTB in both anterior and posterior brain RG-gliomas with labradimil infusion is most likely due to the reduction in blood flow to brain tumor tissue resulting from the fall in MABP caused by the peptide's infusion. Furthermore, since the affinity of labradimil for the bradykinin B2 receptor is lower than that for bradykinin[20,26], we would expect that labradimil would be less potent at increasing the leakiness of the BBTB, and therefore, increases in the transvascular flow rate across the BBTB mediated by labradimil would be overshadowed by the reduction in tumor blood flow.

Although there was an increase in tissue half life of Gd-DTPA in both brain tumor and skeletal muscle, there were clear differences in the pharmacokinetic behavior of Gd-DTPA within these tissues. In the case of brain tumor tissue, the overall shape of the Gd-DTPA concentration curve profiles was consistent with the transvascular extravasation of Gd-DTPA into the extravascular tumor tissue space (Figure 5A through 5E, and Additional file 1). In contrast, in skeletal muscle tissue, the shape of the Gd-DTPA concentration curve profiles always mirrored the respective blood Gd-DTPA concentration curve profile consistent with the retention of Gd-DTPA within skeletal muscle microvasculature, and insignificant extravasation into the extravascular skeletal muscle tissue space (Figure 6A through 6E, and Additional file 2). These findings are consistent with the fact that brain tumor tissue microvasculature is porous[49], while skeletal muscle tissue microvasculature is continuous[29,30]. Therefore, the selectivity of drug accumulation into the extravascular tissue space is governed by the inherent porosity of tissue microvasculature.

When we compared the 1^st ^and 2^nd ^Gd-DTPA concentration curve profiles from blood, brain tumor, and skeletal muscle, it became apparent that the extent of the increase in the half-life of Gd-DTPA within blood, brain tumor, and skeletal muscle correlated with the known metabolic stabilities of the respective bradykinin B2 receptor agonists. Met-Lys-BK and labradimil, were most effective in increasing the half-life of Gd-DTPA, and of the two, labradimil's effect was more significant.

To quantify the effect of the increase in blood half-life of Gd-DTPA on the accumulation of Gd-DTPA in the brain tumor tissue extravascular space, we calculated the percent change in the Gd-DTPA AUC between the 1^st ^and 2^nd ^Gd-DTPA concentration curve profiles of RG-2 glioma brain tumor tissue. When we compared the percent change in Gd-DTPA AUC of each bradykinin B2 receptor agonist group to that of the NS group, we found that there was a statistically significant tumor volume effect on the percent change in Gd-DTPA AUC in anterior brain tumors, but not in the case of posterior brain RG-2 gliomas, which were smaller and had a narrower range of tumor volume distributions than the anterior brain RG-2 gliomas in the study (Additional file 1). In the case of anterior brain RG-2 gliomas, our findings suggest that observed increases in Gd-DTPA AUC due to the systemic infusion of bradykinin B2 agonists are dependent on RG-2 glioma tumor volume and therefore, the transvascular accumulation of Gd-DTPA increases with increasing tumor volume. For anterior brain RG-2 gliomas, there was a statistically significant increase in the Gd-DTPA AUC with the intravenous infusions of Met-Lys-BK and labradimil. Similar trends were noted in the case of the smaller posterior brain RG-2 gliomas although not statistically significant. Being metabolically stable bradykinin B2 receptor agonists, Met-Lys-BK and labradimil increased the blood half-life of Gd-DTPA for sufficiently long to significantly increase the transvascular accumulation of Gd-DTPA into the extravascular brain tumor space. In the case of labradimil, our findings with Gd-DTPA are consistent with those previously reported with carboplatin, as it has been shown that labradimil produces greater increases in the transvascular accumulation of radioactive carboplatin across the BBTB of larger more mature RG-2 glioma brain tumor colonies than across the BBTB of smaller emerging tumor colonies [33].

On analysis of the percent change in skeletal muscle tissue Gd-DTPA AUC, we found there to be significant increases in Gd-DTPA AUC with only Met-Lys-BK and labradimil infusions. Even as such, all bradykinin B2 receptor agonist infusions, including those of BK and Lys-BK, increased the peak Gd-DTPA concentration within skeletal muscle microvasculature, consistent with an increase in blood flow to skeletal muscle due to the vasodilatation of skeletal muscle microvasculature. This would indicate the presence of a "steal effect" due to the shunting of blood flow away from tumor tissue and into skeletal muscle tissue as has been shown in the case of hydralazine[60]. The most important difference between brain tumor and skeletal muscle tissues, in the context of the observed increases in Gd-DTPA AUC of the respective tissues with Met-Lys-BK and labradimil infusions, was that Gd-DTPA remained predominantly intravascular in skeletal muscle tissue due to the continuous nature of skeletal muscle microvasculature.

Based on our dynamic-contrast enhanced MRI-based findings, it is evident that there is an association between the metabolic stability of a bradykinin B2 receptor agonist, the resultant fall in blood pressure during intravenous infusion, and the increase in blood half-life of the co-infused agent, in this Gd-DTPA, a small MRI contrast agent. Here we did not characterize the dose-response relationship for Met-Lys-BK and labradimil, however, in future studies it will be important to do so, to define the systemic parameters necessary for the maximal enhancement in the transvascular delivery of small therapeutics with co-infused metabolically stable bradykinin B2 agonists. The primary mechanism by which metabolically stable bradykinin B2 agonists increase the transvascular delivery of Gd-DTPA across the BBTB of RG-2 gliomas is by increasing the blood half-life of the agent. We were able to establish that the observed increase in the blood half-life of Gd-DTPA results in the increase in transvascular delivery of Gd-DTPA into RG-2 glioma tumor tissue.

We have shown here that metabolically stable bradykinin B2 receptor agonists directly enhance the transvascular delivery of Gd-DTPA by increasing the blood half-life of co-infused small therapeutics. Furthermore, we speculate that metabolically stable bradykinin B2 receptor agonists may increase the blood half-life of co-infused compounds by temporarily decreasing the renal filtration fraction[61], as a result of efferent arteriole vasodilatation[62]. It is also possible that hydralazine, another systemic vasodilator, acts in an analogous manner to increase the effectiveness of co-infused chemotherapy drugs[63,64].

## Conclusion

We found that metabolically stable bradykinin B2 receptor agonists increase the transvascular delivery of small therapeutic and imaging agents across the BBTB of malignant glioma tissue by increasing the blood half-life of the co-infused agent. The selective increase in transvascular delivery across the BBTB of malignant glioma tumor tissue, but not across the continuous microvasculature of normal brain tissue or skeletal muscle tissue, is due to the inherent porous nature of the BBTB of malignant glioma microvasculature.

## Competing interests

The authors declare that they have no competing interests.

## Authors' contributions

HS conceptualized and designed research, performed research, analyzed data, and wrote the manuscript; ASK performed research, analyzed data, and prepared figures; SHF contributed to the experimental design and performed research; JAB contributed to the experimental design; RWC provided dynamic contrast-enhanced MRI analytic tools; DG provided dynamic contrast-enhanced MRI analytic tools; RR provided dynamic contrast-enhanced MRI analytic tools; SA performed statistical analysis.

## Supplementary Material

Additional File 1 **Comparison of the changes in blood and RG-2 glioma tumor tissue Gd concentrations during 15 minute intravenous infusion of normal saline or respective bradykinin B2 agonist**. Additional figure. Error bars represent standard deviation. Click here for file

Additional File 2 **Comparison of the changes in blood and temporalis skeletal muscle tissue Gd concentrations during 15 minute intravenous infusion of normal saline or respective bradykinin B2 agonist**. Error bars represent standard deviation. Click here for file

Additional File 3 **Tumor volumes of anterior brain and posterior brain RG-2 gliomas**. Additional figure. Click here for file

## References

[B1] Wolburg H, Lippoldt A (2002). Tight junctions of the blood-brain barrier: Development, composition and regulation. Vascular Pharmacology.

[B2] Begley DJ, Brightman MW (2003). Structural and functional aspects of the blood-brain barrier. Prog Drug Res.

[B3] Groothuis DR, Vick NA (1982). Brain tumors and the blood – brain barrier. Trends in Neurosciences.

[B4] Brasch RC, Weinmann HJ, Wesbey GE (1984). Contrast-enhanced NMR imaging: animal studies using gadolinium-DTPA complex. AJR Am J Roentgenol.

[B5] Vick NA, Bigner DD (1972). Microvascular abnormalities in virally-induced canine brain tumors. Structural bases for altered blood-brain barrier function. J Neurol Sci.

[B6] Weinmann HJ, Brasch RC, Press WR, Wesbey GE (1984). Characteristics of gadolinium-DTPA complex: A potential NMR contrast agent. American Journal of Roentgenology.

[B7] Roberts HC, Roberts TP, Brasch RC, Dillon WP (2000). Quantitative measurement of microvascular permeability in human brain tumors achieved using dynamic contrast-enhanced MR imaging: Correlation with histologic grade. American Journal of Neuroradiology.

[B8] Larsson HB, Stubgaard M, Frederiksen JL, Jensen M, Henriksen O, Paulson OB (1990). Quantitation of blood-brain barrier defect by magnetic resonance imaging and gadolinium-DTPA in patients with multiple sclerosis and brain tumors. Magnetic Resonance in Medicine.

[B9] Stewart LA (2002). Chemotherapy in adult high-grade glioma: A systematic review and meta-analysis of individual patient data from 12 randomised trials. Lancet.

[B10] Regoli D, Gobeil F, Nguyen QT, Jukic D, Seoane PR, Salvino JM, Sawutz DG (1994). Bradykinin receptor types and B2 subtypes. Life Sciences.

[B11] Regoli D, Barabé J (1980). Pharmacology of bradykinin and related kinins. Pharmacological Reviews.

[B12] Raidoo DM, Sawant S, Mahabeer R, Bhoola KD (1999). Kinin receptors are expressed in human astrocytic tumour cells. Immunopharmacology.

[B13] Liu Y, Hashizume K, Chen Z, Samoto K, Ningaraj N, Asotra K, Black KL (2001). Correlation between bradykinin-induced blood-tumor barrier permeability and B2 receptor expression in experimental brain tumors. Neurol Res.

[B14] Zhao YS, Xue YX, Liu YH, Fu W, Jiang NJ, An P, Wang P, Yang ZH, Wang YQ (2005). Correlation between expression of glioma bradykinin B2 receptor and pathological grade of glioma. Neuroscience Bulletin.

[B15] Wu J, Akaike T, Hayashida K, Miyamoto Y, Nakagawa T, Miyakawa K, Müller-Esterl W, Maeda H (2002). Identification of bradykinin receptors in clinical cancer specimens and murine tumor tissues. International Journal of Cancer.

[B16] Weyerbrock A, Walbridge S, Pluta RM, Saavedra JE, Keefer LK, Oldfield EH (2003). Selective opening of the blood-tumor barrier by a nitric oxide donor and long-term survival in rats with C6 gliomas. Journal of Neurosurgery.

[B17] Emerich DF, Dean RL, Snodgrass P, Lafreniere D, Agostino M, Wiens T, Xiong H, Hasler B, Marsh J, Pink M, Kim BS, Perdomo B, Bartus RT (2001). Bradykinin modulation of tumor vasculature: II. activation of nitric oxide and phospholipase A2/prostaglandin signaling pathways synergistically modifies vascular physiology and morphology to enhance delivery of chemotherapeutic agents to tumors. J Pharmacol Exp Ther.

[B18] Wu J, Akaike T, Maeda H (1998). Modulation of enhanced vascular permeability in tumors by a bradykinin antagonist, a cyclooxygenase inhibitor, and a nitric oxide scavenger. Cancer Research.

[B19] Emerich DF, Snodgrass P, Dean RL, Lafreniere D, Agostino M, Wiens T, Xiong H, Hasler B, Marsh J, Pink M, Kim BS, Bartus RT (2001). Bradykinin modulation of tumor vasculature: I. Activation of B2 receptors increases delivery of chemotherapeutic agents into solid peripheral tumors, enhancing their efficacy. J Pharmacol Exp Ther.

[B20] Emerich DF, Dean RL, Osborn C, Bartus RT (2001). The development of the bradykinin agonist labradimil as a means to increase the permeability of the blood-brain barrier: From concept to clinical evaluation. Clinical Pharmacokinetics.

[B21] Matsukado K, Inamura T, Nakano S, Fukui M, Bartus RT, Black KL (1996). Enhanced tumor uptake of carboplatin and survival in glioma-bearing rats by intracarotid infusion of bradykinin analog, RMP-7. Neurosurgery.

[B22] Barth RF, Yang W, Bartus RT, Moeschberger ML, Goodman JH (1999). Enhanced delivery of boronophenylalanine for neutron capture therapy of brain tumors using the bradykinin analog Cereport (Receptor-Mediated Permeabilizer-7). Neurosurgery.

[B23] Barnett FH, Rainov NG, Ikeda K, Schuback DE, Elliott P, Kramm CM, Chase M, Qureshi NH, Harsh Gt, Chiocca EA, Breakefield XO (1999). Selective delivery of herpes virus vectors to experimental brain tumors using RMP-7. Cancer Gene Ther.

[B24] Emerich DF, Dean RL, Marsh J, Pink M, Lafreniere D, Snodgrass P, Bartus RT (2000). Intravenous cereport (RMP-7) enhances delivery of hydrophilic chemotherapeutics and increases survival in rats with metastatic tumors in the brain. Pharm Res.

[B25] Elliott PJ, Hayward NJ, Dean RL, Blunt DG, Bartus RT (1996). Intravenous RMP-7 selectively increases uptake of carboplatin into rat brain tumors. Cancer Research.

[B26] Bartus RT, Elliott P, Hayward N, Dean R, McEwen EL, Fisher SK (1996). Permeability of the blood brain barrier by the bradykinin agonist, RMP-7: Evidence for a sensitive, auto-regulated, receptor-mediated system. Immunopharmacology.

[B27] Warren K, Gervais A, Aikin A, Egorin M, Balis FM (2004). Pharmacokinetics of carboplatin administered with lobradimil to pediatric patients with brain tumors. Cancer Chemother Pharmacol.

[B28] Thomas HD, Lind MJ, Ford J, Bleehen N, Calvert AH, Boddy AV (2000). Pharmacokinetics of carboplatin administered in combination with the bradykinin agonist Cereport (RMP-7) for the treatment of brain tumours. Cancer Chemotherapy and Pharmacology.

[B29] Bruns RR, Palade GE (1968). Studies on blood capillaries. I. General organization of blood capillaries in muscle. Journal of Cell Biology.

[B30] Trap-Jensen J, Lassen NA (1971). Restricted diffusion in skeletal muscle capillaries in man. Am J Physiol.

[B31] Groothuis DR, Fischer JM, Pasternak JF, Blasberg RG, Vick NA, Bigner DD (1983). Regional measurements of blood-to-tissue transport in experimental RG-2 rat gliomas. Cancer Res.

[B32] Hasegawa H, Ushio Y, Hayakawa T (1983). Changes of the blood-brain barrier in experimental metastatic brain tumors. Journal of Neurosurgery.

[B33] Bartus RT, Snodgrass P, Dean RL, Kordower JH, Emerich DF (2000). Evidence that Cereport's ability to increase permeability of rat gliomas is dependent upon extent of tumor growth: implications for treating newly emerging tumor colonies. Exp Neurol.

[B34] Ferrier MC, Sarin H, Fung SH, Schatlo B, Pluta RM, Gupta SN, Choyke PL, Oldfield EH, Thomasson D, Butman JA (2007). Validation of dynamic contrast-enhanced magnetic resonance imaging-derived vascular permeability measurements using quantitative autoradiography in the RG2 rat brain tumor model. Neoplasia.

[B35] Asotra K, Ningaraj N, Black KL (2003). Measurement of Blood-Brain and Blood-Tumor Barrier Permeabilities with [14C]-Labeled Tracers.

[B36] Andersen C, Taagehøj JF, Mühler A, Rehling M (1996). Approximation of arterial input curve data in MRI estimation of cerebral blood-tumor-barrier leakage: Comparison between Gd-DTPA and 99mTc-DTPA input curves. Magnetic Resonance Imaging.

[B37] Rohrer M, Bauer H, Mintorovitch J, Requardt M, Weinmann HJ (2005). Comparison of magnetic properties of MRI contrast media solutions at different magnetic field strengths. Investigative Radiology.

[B38] Sarin H, Kanevsky AS, Wu H, Brimacombe KR, Fung SH, Sousa AA, Auh S, Wilson CM, Sharma K, Aronova MA, Leapman RD, Griffiths GL, Hall MD (2008). Effective transvascular delivery of nanoparticles across the blood-brain tumor barrier into malignant glioma cells. J Transl Med.

[B39] Inamura T, Black KL (1994). Bradykinin selectively opens blood-tumor barrier in experimental brain tumors. J Cereb Blood Flow Metab.

[B40] Bartus RT, Snodgrass P, Marsh J, Agostino M, Perkins A, Emerich DF (2000). Intravenous Cereport (RMP-7) modifies topographic uptake profile of carboplatin within rat glioma and brain surrounding tumor, elevates platinum levels, and enhances survival. J Pharmacol Exp Ther.

[B41] Haacke EM, Brown RW, Thompson MR, Venkatesan M (1999). Magnetic Resonance Imaging: Physical Principles and Sequence Design.

[B42] Aime S, Nano R (1988). Factors determining the proton T1 relaxivity in solutions containing Gd-DTPA. Invest Radiol.

[B43] Paxinos G, Watson C (2004). The Rat Brain in Stereotaxic Coordinates.

[B44] Cox RW (1996). AFNI: software for analysis and visualization of functional magnetic resonance neuroimages. Comput Biomed Res.

[B45] Lee HB, Blaufox MD (1985). Blood volume in the rat. J Nucl Med.

[B46] Tofts PS, Brix G, Buckley DL, Evelhoch JL, Henderson E, Knopp MV, Larsson HB, Lee TY, Mayr NA, Parker GJ, Port RE, Taylor J, Weisskoff RM (1999). Estimating kinetic parameters from dynamic contrast-enhanced T(1)-weighted MRI of a diffusable tracer: standardized quantities and symbols. J Magn Reson Imaging.

[B47] Wittlich F, Kohno K, Mies G, Norris DG, Hoehn-Berlage M (1995). Quantitative measurement of regional blood flow with gadolinium diethylenetriaminepentaacetate bolus track NMR imaging in cerebral infarcts in rats: validation with the iodo[14C]antipyrine technique. Proc Natl Acad Sci USA.

[B48] Tofts PS, Kermode AG (1991). Measurement of the blood-brain barrier permeability and leakage space using dynamic MR imaging. 1. Fundamental concepts. Magn Reson Med.

[B49] Cox DJ, Pilkington GJ, Lantos PL (1976). The fine structure of blood vessels in ethylnitrosourea-induced tumours of the rat nervous system: with special reference to the breakdown of the blood-brain barrier. Br J Exp Pathol.

[B50] Molnar P, Blasberg RG, Horowitz M (1983). Regional blood-to-tissue transport in RT-9 brain tumors. Journal of Neurosurgery.

[B51] McCarthy DA, Potter DE, Nicolaides ED (1965). An In Vivo Estimation Of The Potencies And Half-Lives Of Synthetic Bradykinin And Kallidin. J Pharmacol Exp Ther.

[B52] Elliott DF, Lewis GP (1965). Methionyl-lysyl-bradykinin, a new kinin from ox blood. Biochem J.

[B53] Straub JA, Akiyama A, Parmar P (1994). In vitro plasma metabolism of RMP-7. Pharmaceutical Research.

[B54] Chan RC, Babbs CF, Vetter RJ, Lamar CH (1984). Abnormal response of tumor vasculature to vasoactive drugs. J Natl Cancer Inst.

[B55] Algire GH, Legallais FY (1951). Vascular reactions of normal and malignant tissues in vivo. IV. The effect of peripheral hypotension on transplanted tumors. J Natl Cancer Inst.

[B56] Rinck PA, Muller RN (1999). Field strength and dose dependence of contrast enhancement by gadolinium-based MR contrast agents. European Radiology.

[B57] Sato H, Enmi J, Teramoto N, Hayashi T, Yamamoto A, Tsuji T, Naito H, Iida H (2008). Comparison of Gd-DTPA-induced signal enhancements in rat brain C6 glioma among different pulse sequences in 3-tesla magnetic resonance imaging. Acta Radiologica.

[B58] Sevick EM, Jain RK (1989). Geometric resistance to blood flow in solid tumors perfused ex vivo: effects of tumor size and perfusion pressure. Cancer Res.

[B59] Henderson E, Sykes J, Drost D, Weinmann HJ, Rutt BK, Lee TY (2000). Simultaneous MRI measurement of blood flow, blood volume, and capillary permeability in mammary tumors using two different contrast agents. Journal of Magnetic Resonance Imaging.

[B60] Belfi CA, Paul CR, Shan S, Ngo FQ (1994). Comparison of the effects of hydralazine on tumor and normal tissue blood perfusion by MRI. Int J Radiat Oncol Biol Phys.

[B61] Clappison BH, Anderson WP, Johnston CI (1981). Renal hemodynamics and renal kinins after angiotensin-converting enzyme inhibition. Kidney International.

[B62] Kon V, Fogo A, Ichikawa I (1993). Bradykinin causes selective efferent arteriolar dilation during angiotensin I converting enzyme inhibition. Kidney Int.

[B63] Bibby MC, Loadman PM, al-Ghabban AF, Double JA (1992). Influence of hydralazine on the pharmacokinetics of tauromustine (TCNU) in mice. Br J Cancer.

[B64] Quinn PK, Bibby MC, Cox JA, Crawford SM (1992). The influence of hydralazine on the vasculature, blood perfusion and chemosensitivity of MAC tumours. Br J Cancer.

