# Supplementary figures and images for: Metabolically stable bradykinin B2 receptor agonists enhance transvascular drug delivery into malignant brain tumors by increasing drug half-life
Source: J Transl Med. 2009 May 13;7:33. doi: 10.1186/1479-5876-7-33 (PMC2689161; doi:10.1186/1479-5876-7-33)

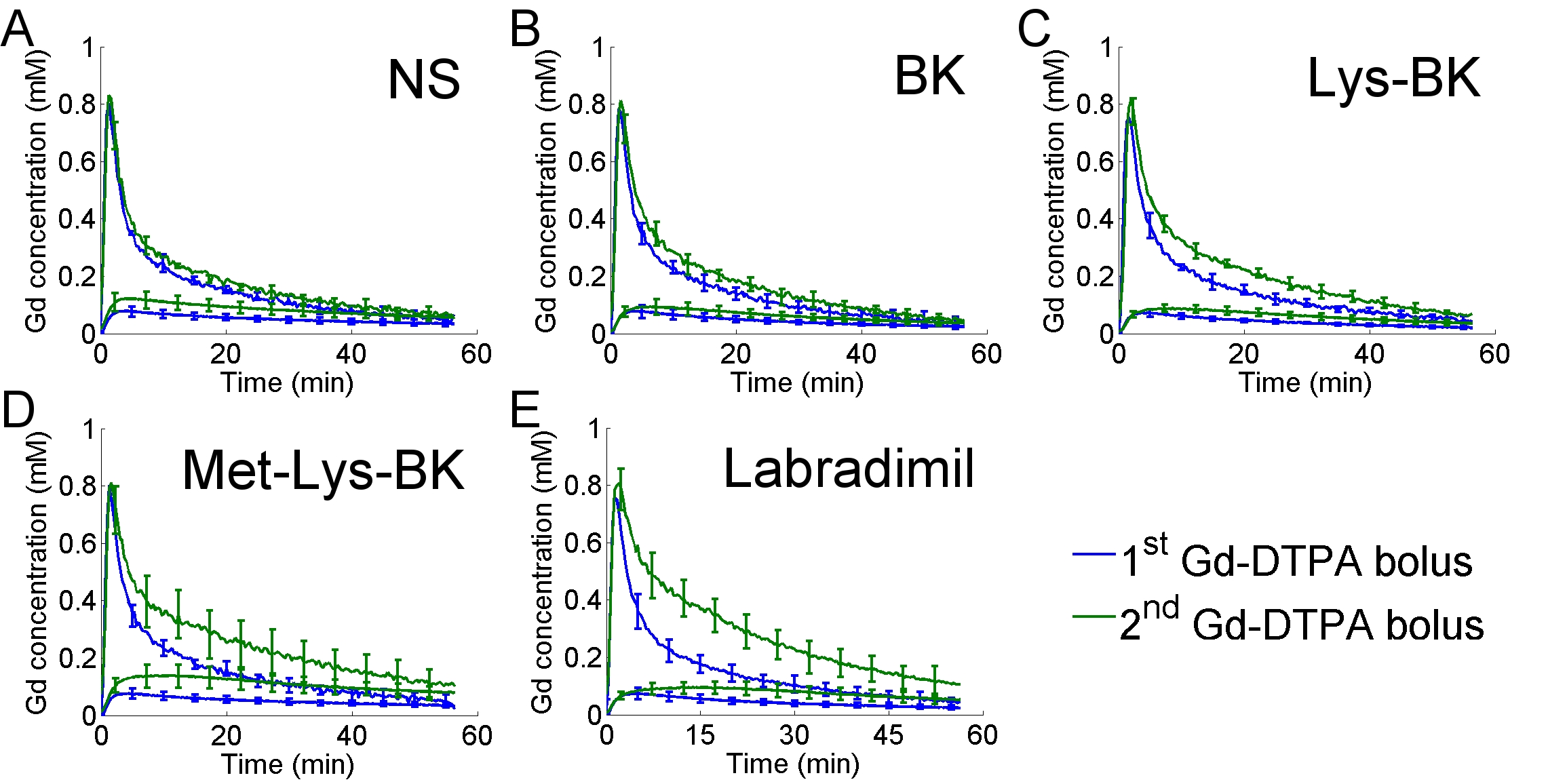

Supplement: Additional File 1 — Comparison of the changes in blood and RG-2 glioma tumor tissue Gd concentrations during 15 minute intravenous infusion of normal saline or respective bradykinin B2 agonist. Additional figure. Error bars represent standard deviation. [file 1479-5876-7-33-S1.jpeg]

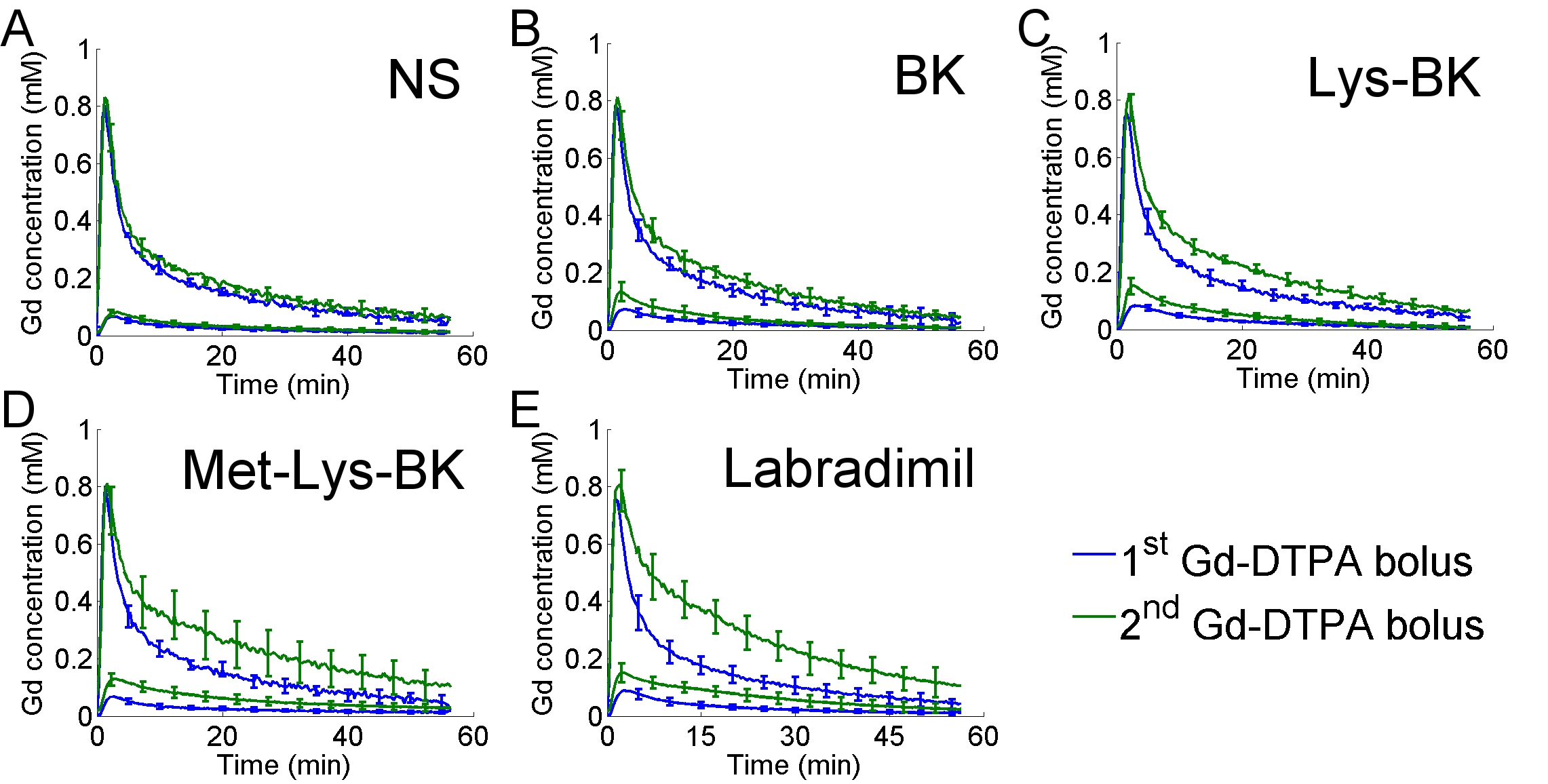

Supplement: Additional File 2 — Comparison of the changes in blood and temporalis skeletal muscle tissue Gd concentrations during 15 minute intravenous infusion of normal saline or respective bradykinin B2 agonist. Error bars represent standard deviation. [file 1479-5876-7-33-S2.jpeg]

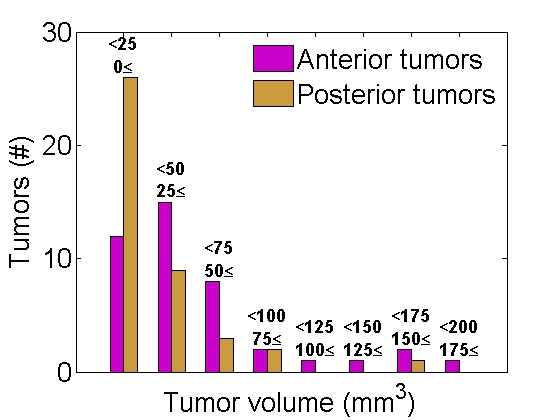

Supplement: Additional File 3 — Tumor volumes of anterior brain and posterior brain RG-2 gliomas. Additional figure. [file 1479-5876-7-33-S3.jpeg]
